# Detection of Urinary Excreted Fungal Galactomannan-like Antigens for Diagnosis of Invasive Aspergillosis

**DOI:** 10.1371/journal.pone.0042736

**Published:** 2012-08-10

**Authors:** Simon F. Dufresne, Kausik Datta, Xinming Li, Ekaterina Dadachova, Janet F. Staab, Thomas F. Patterson, Marta Feldmesser, Kieren A. Marr

**Affiliations:** 1 Johns Hopkins University School of Medicine, Baltimore, Maryland, United States of America; 2 The Sidney Kimmel Comprehensive Cancer Center, Johns Hopkins Medical Institutions, Baltimore, Maryland, United States of America; 3 Albert Einstein College of Medicine, Bronx, New York, United States of America; 4 University of Texas Health Science Center and South Texas Veterans Healthcare System, San Antonio, Texas, United States of America; 5 Département de Microbiologie et Immunologie, Université de Montréal, Montréal, Quebec, Canada; 6 China Medical University, Shenyang, People's Republic of China; Duke University Medical Center, United States of America

## Abstract

Mortality associated with invasive aspergillosis (IA) remains high, partly because of delayed diagnosis. Detection of microbial exoantigens, released in serum and other body fluids during infection, may help timely diagnosis. In course of IA, *Aspergillus* galactomannan (GM), a well established polysaccharide biomarker, is released in body fluids including urine. Urine is an abundant, safely collected specimen, well-suited for point-of-care (POC) testing, which could play an increasing role in screening for early disease. Our main objective was to demonstrate GM antigenuria as a clinically relevant biological phenomenon in IA and establish proof-of-concept that it could be translated to POC diagnosis. Utilizing a novel IgM monoclonal antibody (MAb476) that recognizes GM-like antigens from *Aspergillus* and other molds, we demonstrated antigenuria in an experimental animal IA model (guinea pig), as well as in human patients. In addition, we investigated the chemical nature of the urinary excreted antigen in human samples, characterized antigen detection in urine by immunoassays, described a putative assay inhibitor in urine, and indicated means of alleviation of the inhibition. We also designed and used a lateral flow immunochromatographic assay to detect urinary excreted antigen in a limited number of IA patient urine samples. In this study, we establish that POC diagnosis of IA based on urinary GM detection is feasible. Prospective studies will be necessary to establish the performance characteristics of an optimized device and define its optimal clinical use.

## Introduction

Mortality associated with invasive aspergillosis (IA) is high despite availability of potent antifungal drugs [1]. Delayed diagnosis partly contributes to this poor outcome, since classical microbiological methods are insensitive [2]. Culture-independent detection of several fungal components have shown promise for earlier diagnosis [3]–[6]. Of these, *Aspergillus fumigatus* galactomannan (GM) is a well-studied biomarker [7]–[10]; structurally, it consists of β-(1,5) galactofuranose (Gal*f*) side chains attached to a mannopyranose backbone, forming a part of the cell wall along with Gal*f*-containing glycoproteins and other molecules [11]–[13]. In existing literature, the Gal*f*-containing fungal antigens detected *in vivo* have been collectively referred to as GM, or alternatively, Gal*f* antigens [14], given their immunologically defined nature.

GM is thought to be secreted by growing hyphae, and *in vivo*, bloodstream spillage probably coincides with angioinvasion by the fungus, resulting in detectable antigenemia [14]. Several commercially available immunoassays detecting this antigen have been widely used for almost two decades [7]–[9]. However, its optimal utilization, with regards to appropriate host, specimen type, timing and frequency of sampling, is not entirely defined. Also, new diagnostic challenges have been raised by later timing of infection, usually now after discharge from the hospital [15]. In this setting, point-of-care (POC) testing could have a key role as an aid to diagnose and screen for early disease.

A few studies have provided insight regarding the potential use of urine for antigen detection in IA. The physiology of circulating GM *in vivo* remains largely unknown. Animal data suggest that GM is partly excreted in urine [16], [17]; in humans, urinary excretion of GM was supported immunologically with assays utilizing EBA1 [18] and EBA2 [19], two rat IgM monoclonal antibodies (MAbs) which bind galactofuranosyl residues in GM [20]. However, the exact nature of the Gal*f* antigens excreted in urine and the clinical significance of antigenuria are yet to be determined. In our study, we sought to characterize further the Gal*f* antigens in urine in IA and their detection, using a novel mouse monoclonal antibody, MAb476. We also describe an inhibitory effect of urine on MAb476-based immunoassays and explore methods to overcome the inherent pitfalls associated with testing this complex body fluid. Finally, we report on the development of a lateral flow immunochromatographic assay, amenable to point-of-care testing, detecting urinary Gal*f* antigens in humans with IA.

## Materials and Methods

### Ethics statement regarding animal models and human samples

Mouse experiments were conducted at the Albert Einstein College of Medicine, Bronx, NY, following a protocol approved by the Institutional Animal Care and Use Committee (IACUC) of that institution (Protocol number: 20090404); imaging procedures were performed under isofluorane anesthesia according to protocol. Experiments with the Guinea Pig model of invasive aspergillosis were conducted at the San Antonio Center for Medical Mycology of the University of Texas Health Science Center, following protocols approved by IACUC (Protocol number: 00101G). All studies involving human urine samples were approved by the Johns Hopkins Medicine Institutional Review Board (IRB) (collection of urine from consented human volunteers, study number: NA_00029178; use of previously collected, de-identified, banked urine samples, study number: NA_00027228); written consent forms are in file.

### Fungal strains and exoantigen preparations

Secreted exoantigens were ethanol-precipitated (EP) from mycelial culture supernatants by a slight modification of the method in Latgé *et al.* [11] (**Protocol S1**). EP antigen from *A. fumigatus* strain Af293 grown in *Aspergillus* Minimal Medium (AMM) [21] (henceforth, designated “EPA293”) was used throughout as a surrogate for Gal*f*-containing antigen in immunoassays. In addition, for cross-reactivity studies, Sabouraud's Dextrose Broth (Difco™ SDB, Beckton, Dickinson & Co., Sparks, MD) was used to grow other molds, including *A. fumigatus ΔglfA* (strain lacks UDP-galactofuranosyl mutase; produces no Gal*f*) and its parental strain D141 (both kind gifts from Dr. Françoise Routier [22]), *A. fumigatus* strain Af293; *Rhizomucor pusillus* (ATCC46342; ATCC, Manassas, VA); and other clinical and environmental isolates (**Table 1**) from our collection [23], as well as yeasts (*Candida albicans* and *Cryptococcus neoformans*). Some *Aspergillus* spp. strains, *Trichophyton rubrum* and *Wangiella dermatitidis* were grown in AMM. Respective EP antigens were prepared as above. The carbohydrate contents of all the lyophilized EP antigens were estimated by a phenol-sulfuric acid microplate method [24]. *Histoplasma capsulatum* (Thon strain) C antigen (C-Ag) purified from mycelial culture filtrate was a kind gift from Dr. Tom Chiller (Centers for Disease Control and Prevention, Atlanta, GA).

**Table 1:** MAb476 cross-reactivity with other fungi^a^.

REACTIVE^b^	NON-REACTIVE
*Aspergillus fumigatus* (Af293 and D141)	*Aspergillus fumigatus (ΔglfA)*
*Aspergillus lentulus* [3]	*Aspergillus terreus* [3]
*Neosartorya udagawae* [4]	*H. capsulatum* C-Ag
*Neosartorya pseudofischeri* [2]	*Scedosporium sp.* [2]
*Aspergillus flavus*	*Alternaria sp.* [3] c
*Aspergillus neoellipticus*	*Rhizopus microsporus*
*Aspergillus nidulans* [2]	*Rhizopus oryzae*
*Aspergillus niger* [2]	*Mucor sp.*
*Aspergillus ustus* [2]	*Rhizomucor pusillus*
*Aspergillus versicolor*	*Absidia corymbifera*
*Fusarium sp.* [2]	*Cunninghamella* sp. [2]
*Paecilomyces sp.*	*Penicillium oxalicum* [2]
*Trichophyton rubrum*	*Scopulariopsis sp.* [2] c
	*Acremonium sp.* [2]
	*Wangiella dermatitidis*
	*Candida albicans*
	*Cryptococcus neoformans*

Notes:

a All antigen preparations were adjusted to 10 µg/ml of carbohydrate content.

b Number of isolates tested indicated in brackets (one isolate if no indication).

c One isolate was weakly reactive.

### Monoclonal antibody

MAb476 is a murine monoclonal IgM with κ light chains (*M. Feldmesser, unpublished data*). The hybridoma was generated using spleen cells from a mouse immunized with germinating *A. fumigatus* conidia and screened for serum reactivity against purified GM.

### Enzyme-linked immunosorbent assays (ELISAs)

For assay details, see **Protocol S2**. Briefly, *indirect* ELISAs (iELISA) were performed on plates coated with EPA293 in PBS and incubated with dilutions of MAb476 in appropriate diluents (per assay design). Binding was detected with Alkaline Phosphatase (AP)-conjugated goat anti-IgM (SouthernBiotech Inc, Birmingham, AL). *Sandwich* ELISAs (sELISA) were performed on plates coated with MAb476 (capture antibody) and incubated with antigen-containing samples in appropriate diluents (per assay design). Antigen detection was done with biotinylated- or AP-conjugated MAb476; biotinylated MAb476 was additionally detected with streptavidin-AP (SouthernBiotech). Variations of sELISA format to test different parameters are described in the corresponding results sections. For animal and human samples, an absorbance of mean of background well values plus 3SD was considered the cutoff for positive.

### MAb476 cross-reactivity

EPs from 49 isolates (18 different genera), as well as *H. capsulatum* C-Ag, were adjusted to 10 µg/ml in PBS (GIBCO®, Life Technologies, Grand Island, NY) and tested for reactivity with MAb476 using sELISA.

### Urine inhibition experiments

Inhibitory effect of urine on MAb476 solid-phase immunoassays was demonstrated in both iELISA (MAb476 serially diluted in urine; detection reagent in PBS/blocking buffer) and sELISA (EPA293 antigen or MAb476-conjugate diluted in urine). Urine was used untreated or treated as specified in the following section. To understand the mechanism of urine inhibition of MAb476 immunoassays, we separately tested urine effect on different immunoassay components/steps (coating, antigen/antibody) as described in Results, by adding extra urine incubation steps (performed at 37°C for 1 h). Urine-incubated MAb476 or EPA293 was buffer exchanged into PBS with a desalting column (see below) before use in immunoassays.

### Characterization of urine inhibitory substances

To determine the molecular size of the inhibitor, four different exclusionary treatments were applied to urine: desalting through 7 KDa or 40 KDa molecular weight cutoff (MWCO) Zeba Spin Columns, or dialysis using 2 KDa or 3.5 KDa MWCO Slide-A-Lyzer® cassettes (both from ThermoFisher). To further identify the chemical nature of the inhibitor, we tested several chemical treatments on urine (10 m boiling followed by centrifugation; alkalinization (pH 8) with sodium hydroxide; acidification (pH 5) with glacial acidic acid; and addition of 5, 10 or 20 mM EDTA), and used the treated urines as EPA293 diluents in sELISA. In addition, we tested known chaotropic molecules present in urine, such as urea and guanidine (guanidine hydrochloride), and divalent cations (known kosmotropes) calcium (Calcium chloride) and magnesium (Magnesium chloride), individually or combined in PBS (pH 6, 7.2 or 7.4) at physiological or supra-physiological levels (final concentration ranges: 50–800, 1–8, 1–20 and 1–20 mmol/L, respectively) [25]–[27]. All chemical reagents used were from Sigma-Aldrich, St. Louis, MO.

### Processing of urine samples

Concentration and desalting/dialysis were tested as possible means to increase assay sensitivity by concentrating the analyte and removing the inhibitor, respectively. To assess effect of concentration on its inhibitory potential, Urine concentrated 5–10 fold with different Amicon® Ultra spin concentrator units (3 or 5 KDa; Millipore Co., Billerica, MA), the spin flow-through, as well as unprocessed urine, were assayed as EPA293 diluents in the sELISA. Urine samples spiked with EPA293 were used as simulated samples and treated by concentration and/or dialysis/desalting; samples were tested before and after treatments using sELISA and LFD. Ten-fold concentration (3 KDa Amicon) followed by desalting (7 KDa spin column) was selected for processing animal and clinical urine samples.

### Experimental animal models of invasive aspergillosis

Briefly, serum, broncho-alveolar lavage fluid (BAL) and lungs were collected, at day 2 post-aerosol infection, from *A. fumigatus* (strain 90906)-infected neutropenic C57BL/6 mice (as described in [28]); serum, BAL and lung homogenates were boiled with 4% EDTA to remove proteins and dissociate preformed antigen-antibody complexes, and assayed for *Aspergillus* antigen in an sELISA. Another set of infected mice were injected intraperitoneally or intravenously with ^99m^Technetium-labeled MAb476 at day 2 post-infection; scintillation imaging was performed under anesthesia 3 h later using methodology described in [29]. In addition, urine samples were collected from immunosuppressed Hartley Guinea Pigs (GP), infected via an inhalational challenge with aerosolized Af293 conidia as described elsewhere [30], at days 0, 3, 5 or 7 post-infection. Samples with sufficient volume were assayed by sELISA. GP serum GM was tested with the commercial galactomannan-enzyme-immunoassay (“GM-EIA”; Platelia™ *Aspergillus* EIA, BioRad Inc., Hercules, CA).

### Human samples

Urine samples were obtained from 6 healthy adult volunteers (numbered VU1-6). Basic physicochemical parameters were tested with urinalysis strips (Multistix® 10 SG, Siemens Healthcare Diagnostics Inc., Tarrytown, NY). Most experiments involving normal urine were done with samples pooled from 2–6 individuals. One clinical sample from a patient with probable IA [31] was collected with consent at Johns Hopkins Hospital. We also used banked (−80°C) urine samples from a study conducted at Fred Hutchinson Cancer Research Center, Seattle, WA; patients participating in this study, all diagnosed with IA, had urine collected every 8 h for 3 consecutive days. Of these, we selected urine samples from 10 patients (proven/probable IA) with GM-EIA positive serum.

### Characterization of urinary antigen in patients with IA

Urine from two patients with detectable antigenuria was used for characterization of the antigen. Periodate oxidation (Sodium *meta*-Periodate, ThermoFisher) and pronase digestions (*Streptomyces griseus* Pronase, Roche Diagnostics, IN) were performed following manufacturer's instructions. Ethanol precipitation of possible *Aspergillus* exoantigen in urine was done as described for culture supernatant and dialyzed/desalted at different MWCO. All conditions were assayed in the sELISA.

### Development of the lateral flow immunochromatographic assay

For assay details, see **Protocol S3**. Briefly, unconjugated MAb476 and a goat anti-mouse IgM (SouthernBiotech) were immobilized at the test and control spots, respectively, on HF180 Nitrocellulose (NC) membrane (Millipore) strips followed by incubation with the blocking buffer, and colloidal Gold-conjugated MAb476 was applied to blocked conjugate pads. The components were enclosed in a plastic cassette with a sample and a reaction window to fabricate the lateral flow device (LFD). Sample (130–150 µl) was applied dropwise to the sample window; results (spots) were read visually, and scanned in for digital storage.

### Statistical analysis

The following statistical analyses were employed (Prism v.6, GraphPad Software, La Jolla, CA): unpaired Student's *t*-test for comparison of means between two groups; One-way ANOVA with a Tukey's post test for pair-wise comparisons for multi-group comparisons; nonlinear Sigmoidal dose-response (variable slope) regression curves for ELISA results; estimation of concentration to reach half maximal signal (EC_50_) values, computed after normalization of Y-values to 100% and compared with an Extra Sum-Of-Squares F-test; Spearman's correlation test to analyze possible relationship between specific gravity and EC50 of urine ELISA curves. For all comparisons, *p*<0.05 was considered significant, and 0.05>*p*>0.1, a trend towards significance.

## Results

### *In vivo* GM detection and MAb476 localization in a murine model of IA

**Figure 1:**
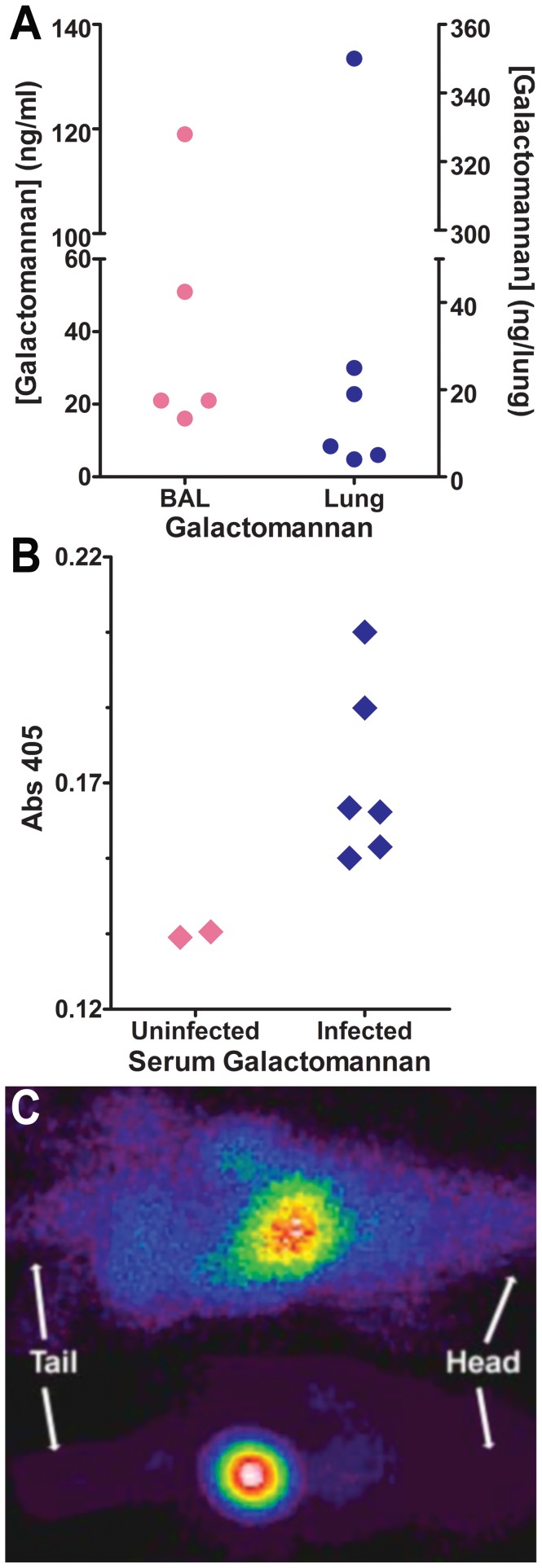
MAb476 recognizes *in vivo* Gal*f* antigen in a murine model of IA and localizes in the bladder of infected mice. *A*,*B* Sandwich ELISA detection of Gal*f*-containing antigen in BAL and lung homogenates (panel A; N = 5–6) and serum (panel B; N = 2–6) of neutropenic mice, 2 days after aerosol infection with *A. fumigatus*. *C*, Organ distribution of ^99m^Tc-MAb476 2 days after infection; *Top*, control mouse: ^99m^Tc-label localization in liver and spleen; *Bottom*, infected mouse: ^99m^Tc-label localized in the bladder, representative of 2 independent experiments (N = 3 per group).

Of several GM-reactive MAbs identified during screening, MAb476 was selected for further investigations because of its higher relative binding affinity compared to four other GM-reactive MAbs (*M. Feldmesser, unpublished data*). In the murine model of pulmonary IA, MAb476 was reactive by sELISA with BAL, lung homogenate, and serum (**Figure 1A,B**) from all infected mice, suggesting recognition of an *in vivo* secreted GM-like antigen. Interestingly, MAb476 radiolabeled with ^99m^Tc (to explore its use in diagnostic imaging) revealed early localization of the radioisotope in the bladder of aerosol-infected animals (**Figure 1C**), with the same pattern seen regardless of whether the radiolabeled MAb was injected intraperitoneally or intravenously (not shown). In contrast, in sham-infected controls, the radiolabeled MAb was mostly distributed in liver and spleen. We hypothesized that the observed rapid bladder localization of MAb476 in infected mice was driven, at least in part, by renal elimination of its cognate antigen, *albeit* through an unclear mechanism. These data suggested that MAb476 could recognize a urinary excreted antigen in IA, and therefore, be used in immunoassays performed on urine. This concept became the main focus of our further investigations.

### Urine inhibition of MAb476 binding

**Figure 2:**
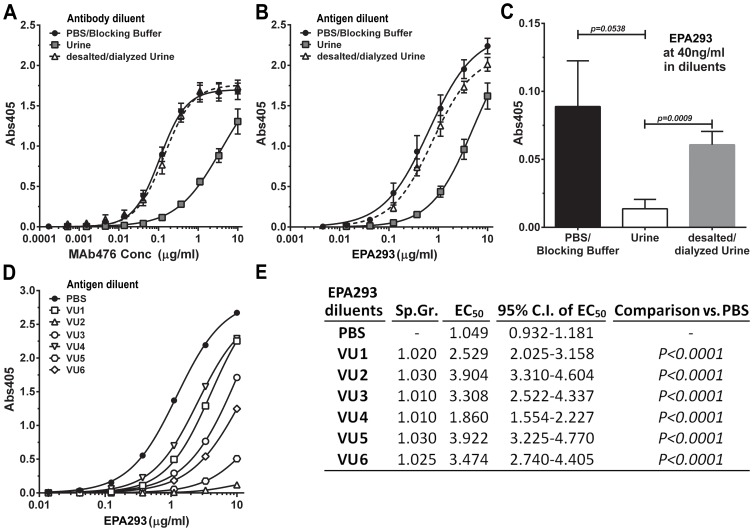
Urine has an inhibitory effect on MAb476-based immunoassays, which is abrogated by desalting or dialysis. *A*, Comparison amongst PBS/blocking buffer, urine and desalted/dialyzed urine as a diluent for the secondary antibody in indirect ELISA. Antibody concentration to reach half maximal signal (EC_50_) in urine is significantly different than in PBS/blocking buffer or in desalted/dialyzed urine (*p*<0.001, F-test; data combined 3–9 independent assays). *B,C*, Comparison of PBS or blocking buffer vs. urine or desalted/dialyzed urine as a diluent for the EPA293 in sELISA. *Panel B:* Antigen concentration to reach half maximal signal (EC_50_) in urine is significantly different than in PBS/blocking buffer or in desalted/dialyzed urine (*p*<0.001, F-test; data combined 9–15 independent assays). *Panel C:* Comparison of mean signals at 40 ng/ml of EPA293 (urine vs. PBS/Blocking buffer and desalted/dialyzed urine, p = 0.0538 and 0.0009, respectively, unpaired *t*-test; N = 7–11 independent assays). *D,E*, Inhibitory effect of urine from 6 healthy volunteers in sELISA; EC_50_ values of all urines were significantly different from that of PBS (*p*<0.0001, F-test).

In MAb476 immunoassays, we compared reactivity to EPA293 with either the antibody (iELISA; **Figure 2A**; N = 3–9) or the antigen (sELISA; **Figure 2B**; N = 9–15) diluted in urine, desalted/dialyzed urine or in PBS/blocking buffer. In both assay formats, we observed a significant diminution of assay signal (absorbance values) in urine (Urine vs. PBS/Blocking buffer, EC_50_ comparison; *p*<0.001, F-test) suggesting that it had an inhibitory effect, which was nearly abrogated by dialysis/desalting through 7 KDa or 40 KDa MWCO devices. Similar observations (**Figure 2C**; Comparison of mean signal; urine vs. PBS/Blocking buffer and desalted/dialyzed urine, p = 0.0538 and 0.0009, respectively, unpaired *t*-test; N = 7–11 independent assays) were made when comparing signal at low EPA293 concentrations, that are biologically relevant to human antigenuria (∼40 ng/ml) [16]. Interestingly, similar inhibition was observed, but to varying degrees, in the healthy volunteer urine samples VU1-6 (**Figure 2D,E**). We confirmed the inhibition phenomenon in LFD assays (shown below).

In attempts to reveal the mechanism of urine inhibition of MAb476 immunoassays, we tested several variables. Urine incubation of MAb476-coated and blocked wells, prior to antigen addition, had no effect on the signal (or increase in background absorbances), indicating that urine did not affect immobilized MAb476 or interfere with blocking (**Figure S1A**). Also, pre-incubation of MAb476 in urine did not affect the physical integrity of IgM MAb476, as assessed by a non-reducing SDS-PAGE (data not shown), or the antigen-binding site, as assessed by a subsequent iELISA on EPA293 coated plates (**Figure S1B**). Similarly, incubation of EPA293 with urine prior to sELISA showed no deleterious effect on its recognition (**Figure S1C**). Therefore, urine's inhibitory effects likely influenced nascent antigen-MAb476 interactions, which would lead to signal diminution when antigen is diluted in urine (**Figure 2B**, Urine). In addition, a considerable reduction in signal was observed whenever a urine incubation step was introduced after any antigen-antibody complexation step. Interestingly, addition of urine after formation of a [capture-MAb476]-antigen-[MAb476-conjugate] supercomplex (following MAb-conjugate step) showed worse outcome (EC_50_ comparison, *p* = 0.0058, F-test) in comparison to urine after formation of [capture-MAb476]-antigen complexes (following EPA293 incubation step), which likely reflects urine's additive effect in disruption of all preformed bonds between antigen and MAb476 (**Figure S1D**). Taken together, these observations indicated that urine negatively impacts all stages of antigen-MAb476 interactions, which was corroborated by the occurrence of maximum diminution of the signal when the MAb476-conjugate is diluted in urine (data not shown). This phenomenon has particular significance for the development of a urine-based LFD where antigen-antibody interactions would occur sequentially in mobile/solution and immobile phases.

We investigated the putative ‘urine inhibitor’. Of note, specific gravity (Sp. Gr.) of urine samples from healthy donors (**Figure 2E**) was significantly positively correlated (Spearman *ρ* = 0.8827, *p* = 0.044) with EC_50_. This suggested the inhibitor concentration as an important parameter. Our observations (also see **Dataset S1**) indicated that (a) dialysis/desalting through all MWCO devices, down to 2 KDa, abrogated the inhibitory effect of urine similarly (**Figure S2A**); (b) concentration of urine did not worsen its inhibitory effect, likely due to passage of the inhibitory molecules through 3–5 KDa MWCO membranes of the concentrator (data not shown); (c) acidification, alkalinization, EDTA treatment, or boiling/centrifugation of urine did not alleviate inhibition, suggesting that the inhibitor was a small (<2 KDa) molecule, not related to inherent acidic pH of normal urine (and not influenced by alkalinization), not likely a divalent cation, and not protein in nature (**Figure S2B**). Interestingly, at supra-physiological concentrations [25]–[27], a mix of chaotropic molecules (urea, guanidine) and common ions in urine was significantly inhibitory as an EPA293 diluent (PBS vs. Mix, EC_50_ comparison; *p* = 0.0005, F-test) (**Figure S2C**). The significance of this observation is as yet unclear, but may suggest that a combination of solutes, and not a single molecule, effect the inhibition.

**Figure 3:**
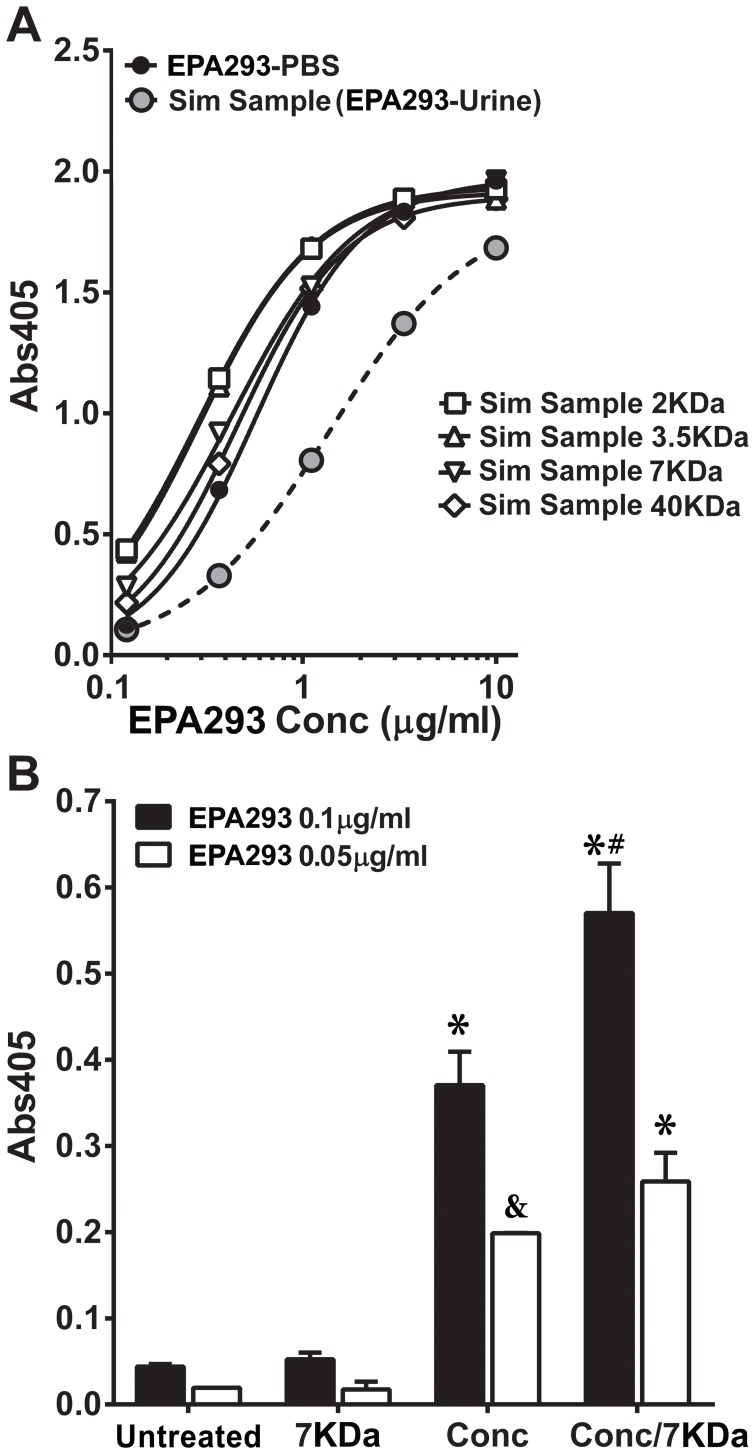
Desalting/dialysis and/or concentration improve antigen detection in simulated urine samples in sELISA. *A*, Detection of EPA293 in simulated (spiked) samples after desalting or dialysis at different MWCOs (40 KDa, 7 KDa, 3.5 KDa and 2 KDa); signal curves in PBS or in all treated urine were significantly (comparison of EC_50_; *p*<0.0001, F-test) different than in untreated urine. *B*, Detection of low, but biologically plausible (50 and 100 ng/ml), EPA293 concentrations in simulated samples after desalting (7 KDa), concentration (5–10 folds; 3 KDa spin-column), or both, compared to no treatment. (*) represents comparisons between a treatment and untreated urine with significant difference (*p*<0.01); (&) represents a trend (*p* = 0.0613) in comparison between a treatment and untreated urine; (#) represents comparison between concentration/desalting and concentration-only treatments with significant difference (*p* = 0.0077); all comparisons by One-way ANOVA with a Tukey's post-test for pair-wise comparisons; N = 2–3.

Simulated clinical samples were prepared by adding EPA293 to urines from healthy volunteers. Dialysis or desalting improved the signal at all MWCOs (comparison of EC_50_; *p*<0.0001, F-test), with apparent antigen retention (**Figure 3A**). Although all MWCO systems used were equally effective in removing the inhibitor, the lowest MWCO (7 KDa) available in a convenient column format was preferred to desalt urine samples. Comparison of untreated vs. treatments of simulated samples containing low, but biologically plausible, analyte concentrations (viz. 50 and 100 ng/ml) (**Figure 3B**) indicated that: (a) desalting alone was ineffective at low antigen concentrations; (b) concentration (5–10fold) of simulated urine samples improved EPA293 detection (*p*<0.0001 and  = 0.0613, respectively, for EPA293 100 and 50 ng/ml; One-way ANOVA/Tukey's post-test; N = 2–3); (c) desalting following concentration further increased the signal at 100 ng/ml significantly (*p* = 0.0077), but not at 50 ng/ml. This combined processing was used to process animal and clinical samples for both ELISA and LFD assays (see below).

### Detection of a urinary antigen in a GP model of IA

**Figure 4:**
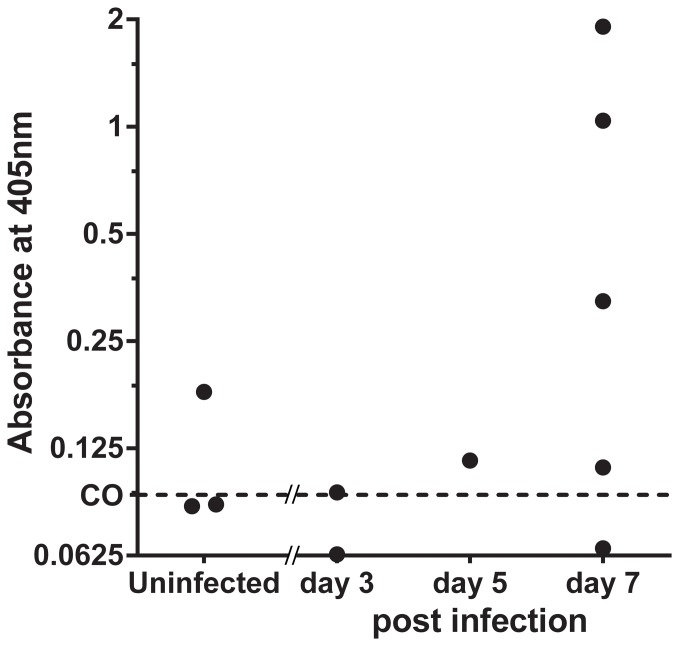
MAb476 detects a urinary excreted Gal*f* antigen in a GP model of pulmonary IA. Processed (10-fold concentration/7 KDa-desalting) urines samples were tested in sELISA; 6 out of 8 infected guinea pigs had detectable antigenuria. Cut off (CO) value equals the mean+3SD of the absorbances of negative controls.

Urine from experimentally infected GP was tested by sELISA after the combination processing of concentration and desalting (**Figure 4**). Antigen was detected in the urines of animals collected at day 3 (1/2), 5 (1/1) and 7 (4/5) post-infection. All GP urines collected at days 5 and 7 were positive for antigenemia as assessed by GM-EIA (data not shown). Surprisingly, one of the three uninfected animals had a positive signal on LFD, and also tested positive by GM-EIA. Together the findings corroborated the hypothesis that the Gal*f* antigen recognized by MAb476 was excreted in urine *in vivo* in this experimental animal model of IA.

### Detection of urinary antigen in humans with IA

**Figure 5:**
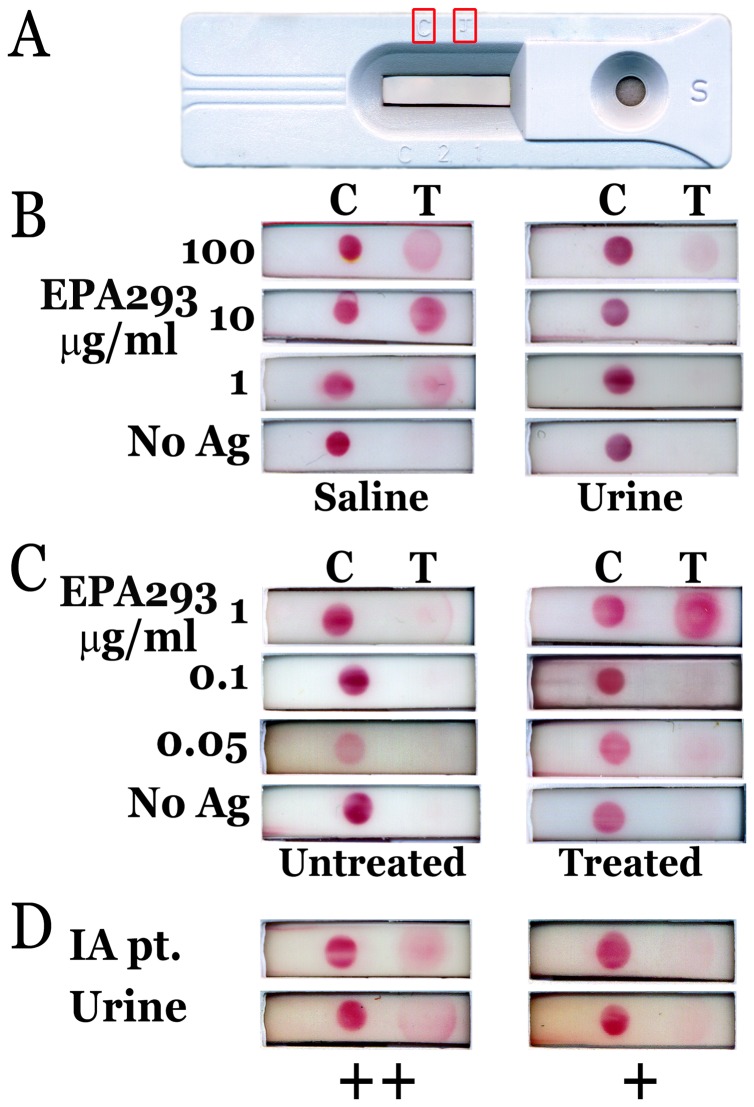
MAb476-based Lateral flow immunochromatographic assay device (LFD) detects Gal*f* antigen in simulated and human urine samples. *A*, appearance of the assembled LFD; subsequent panels show scanned images of the reaction window. *B*, Comparison of urine and normal saline (NS) as diluent for EPA293 in LFD. *C*, Detection of EPA293 in simulated samples after concentration (5–10 folds, 3 KDa MWCO) and desalting (7 KDa MWCO), compared to no treatment. *D*, Detection of Gal*f*-containing antigen in processed clinical urine samples; 2 patients have intermediate (++) positive signal and 2 have weak (+) positive signal.

We assembled in-house an LFD (**Protocol S3;** **Figure 5A**) to test for antigenuria in clinical and animal samples. We confirmed the urine inhibition phenomenon (observed in ELISA) via LFD (**Figure 5B**). The combination processing of concentration and desalting allowed for antigen detection by LFD in simulated urine samples (**Figure 5C**), as well as in clinical samples. Out of 11 clinical samples tested, 4 had detectable antigen by sELISA after processing (data not shown). All 4 samples also were positive by LFD (2 intermediate, 2 weak) (**Figure 5D** **)**.

The exact nature of Gal*f* antigens in human is unknown, which may have important repercussions for assay optimization including sample processing. Therefore, we attempted to partially characterize the urine-excreted antigen utilizing the urine from two patients with antigenuria. Periodate oxidation of concentrated urine from one patient reduced the signal by >90% (confirming its carbohydrate nature), whereas pronase digestion had no significant effect, nor did boiling and centrifuging (data not shown). The urinary antigen was at least partly ethanol precipitable. Ethanol precipitate from urine (EP-U) of two patients was used for further size estimation by dialyzing or desalting with different MWCO devices. For EP-U from one patient, desalting via 40 KDa (but not other MWCOs) resulted in a loss (∼25%) of signal, whereas the other EP-U showed a ∼35% loss of signal at all MWCO (data not shown), indicating wide size-variability of the excreted antigen in humans.

### Characterization of MAb476 specificity

Analytical specificity of MAb476-sELISA was determined using GM-like exoantigens secreted by other Aspergilli and other molds. MAb476, tested against EPs from 49 fungal isolates **(** **Table 1** **)**, bound EP from members of *Aspergillus* Fumigati complex and other *Aspergillus* species, except *A. terreus*. Unlike its parental strain (*A. fumigatus* D141), the *A. fumigatus* Δ*glfA* produced a very weak reaction. Among other hyalohyphomycetes, only *Fusarium* spp., *Paecilomyces* sp., and *T. rubrum* reacted significantly. No zygomycetes (*Absidia corymbifera*, Cunninghamella spp., Mucor sp., *Rhizomucor pusillus*, and *Rhizopus* spp.), phaeohyphomycetes (*Alternaria* spp. and *Wangiella dermatitidis*), or yeasts (*C. albicans* and *C. neoformans*) produced an exoantigen recognizable by MAb476. Purified *Histoplasma capsulatum* C-Ag did not bind MAb476.

## Discussion

This study describes the detection of a urinary excreted Gal*f* antigen by ELISA and LFD using a novel MAb raised to *A. fumigatus* germinating conidia. Observation of an assay inhibitor in urine encourages further investigations towards eventual development of a clinically useful POC assay.

In 1978, Lehmann and Reiss first reported indirect evidence of urinary excretion of GM in a rabbit model of experimental systemic aspergillosis; a GM-reactive antiserum detected antigen in urine and serum from *A. fumigatus*-infected rabbits by counter-immuno-electrophoresis [32]. Dupont and Bennett later confirmed that GM was detectable in urine from both rabbits and humans with IA [16], and that circulating GM was removed from bloodstream through renal excretion in rabbits, recovering approximately 35% of injected radiolabeled GM in urine after 24 h [17]. Using anti-GM rat MAb EBA1, Haynes *et al.* reported a Gal*f*-containing glycoprotein in urine from a few patients with IA [18]. Finally, a limited number of patients with IA were shown to have a detectable antigen in urine by latex agglutination [19], and EIA [8], [33], based on another rat IgM, MAb EBA2. More recently, our studies confirmed detectable antigen in urine in children with IA (Fisher *et al.*, *in press*, 2012). However, these pivotal studies did not address some important questions. First, renal elimination of circulating GM during natural IA was not definitely demonstrated. Indeed, the above-mentioned animal models, showing high-level antigenuria, had significant kidney fungal burden as a result of intravenous conidia delivery. Similarly, many humans with antigenuria had kidney involvement found at autopsy [19]. Secondly, although urine itself as a vehicle was found to hinder antigen detection in some immunoassays [16], [19], [34], this inhibitory effect was never fully described. Finally, a chemical description of the urinary antigen was not reported in humans. The observation that our MAb could specifically recognize a urinary excreted antigen in IA prompted our study, in order to address the remaining questions regarding the detection of *Aspergillus* antigenuria.

We detected an *A. fumigatus* urinary antigen utilizing immunoassays based on a novel murine MAb. A dramatic reduction in binding after mild hydrolysis with HCl (*M. Feldmesser, unpublished data*) suggested that the Gal*f* lateral chains are an essential component of the GM epitope recognized by MAb476, a finding corroborated by the absence of binding with a Gal*f*-deficient strain of *A. fumigatus*. In addition, all tested fungi lacking the Gal*f* moiety (zygomycetes and phaeohyphomycetes) were non-reactive, whereas *Fusarium spp.* and Paecilomyces sp., containing β(1→5) and β(1→6) linked D-galactofuranose [35], were reactive to MAb476. Interestingly, *T. rubrum*, which does contain GM with Gal*f* connected to a mannan backbone but no polygalactofuranosyl chain (as in *A. fumigatus*) [35], [36], was also recognized by MAb476, suggesting that the cognate epitope may not be restricted to the Gal*f* moiety. However, MAb476 appeared not to recognize non-mannan linked α(1→6) Gal*f* molecules present in the secreted galactoxylomannan antigen from *C. neoformans* [37], implying the requirement for specific arrangement or conformation of the Gal*f* moiety for MAb476-recognition. The specificity profile with aforementioned fungi parallels some observations published with EBA2, the commercial GM EIA (Platelia™) MAb [20]. However, three isolates of *A. terreus* were not reactive with MAb476, while detection of *A. terreus* antigen with Platelia™ has been reported [38]–[40]. *A. terreus* complex is a diverse group of organisms; therefore, this discrepancy may represent a difference in species or strains tested [41], or a divergence in epitopes recognized by the antibodies. Similarly, MAb476 did not bind to *H. capsulatum* C-Ag, a surrogate for the *Histoplasma* polysaccharide antigen (HPA) [42], whereas EBA2-based Platelia™ is cross-reactive both in the context of histoplasmosis [43] and to purified C-Ag (unpublished data). Taken altogether, these observations suggest that EBA2 and MAb476 likely recognize different epitopes. In addition, the observed cross-reactivity of MAb476 with multiple fungal pathogens may be clinically useful, as assay positivity would appropriately prompt antifungal therapy in non-*A. fumigatus* invasive mold infections.

In the present work, antigenuria was shown in experimental inhalational models of IA in mice and guinea pigs, as well as in diagnosed IA in humans. In the murine model, the post-renal appearance of the IgM MAb was interesting, since ordinarily large proteins may not pass through the glomerular filter; however, recent evidence suggests that IgM may be present in human urine, even in healthy subjects [44], [45]. Also, in contrast to other models involving intravenous injection of conidia, dissemination of the organism to the kidneys does not appear to occur in the inhalational model; fungal burden remains undetectable in the kidneys by real-time PCR, GM EIA and culture [30]. Antigenuria was detected mainly in animals with antigenemia, supporting the hypothesis of renal elimination of GM circulating in the bloodstream. Among the 11 patients with proven/probable IA, antigenuria was detected in 4. In the remaining 7 samples, GM levels may have been under the detection threshold of our assay or processing may have caused antigen loss in those samples. Alternatively, long-term storage and repeated freeze-thaw cycles may have altered the samples. Sampling time and host factors (e.g. antifungal use, renal failure, etc.) could not be assessed as clinical data were not available.

The finding of an inhibitory effect of urine is an important finding because it affects the use of neat urine for the detection of GM and potentially other polysaccharides. This body fluid is ideal for point-of-care testing and could be of considerable utility in diagnosis of IA. Importantly, the urine inhibitory effect was particularly apparent in the LFD where, in contrast to ELISAs, all antibody-antigen interactions occur in the solution-phase with the specimen as a solvent. The urine inhibition could not be alleviated by boiling and centrifuging, indicating that the culprit was likely not a protein. We also found that the interfering substance was smaller than 2 KDa. Furthermore, there seemed to be a proportional relation between specific gravity and inhibitory effect. We hypothesized that the inhibitor is one or a combination of small molecules with chaotropic properties, which would be more or less concentrated in the urine across individuals. This could account for our observation of varying degree of inhibition with different urine samples. However, its identity remains unknown. Simple procedures such as desalting or dialysis can effectively remove it and restore immunoassay efficacy, while preserving the antigen, potentially allowing device optimization.

The chemical nature of the urinary excreted antigen was assessed using urine from an IA patient. Sensitivity to periodate oxidation and resistance to pronase digestion suggest that the urinary antigen epitope recognized by MAb476 is a carbohydrate. These findings do not formally exclude a glycoprotein bearing an immunoreactive carbohydrate, as has been described for some EBA2-reactive antigens [46]. However, boiling and centrifugation had no effect on epitope recognition, suggesting a non-protein antigen. Also, attempts to identify a glycoprotein immunoreactive with MAb476 were not successful (not shown), although low abundance of the molecule may not be ruled out. In a rabbit model, Dupont *et al.* found that the size of urinary excreted GM had a wide spectrum between 70 KDa and <10 KDa, with most of the antigen approximating 18 KDa [16]. Consistent with those findings, no precise size cut off could be established using the ethanol precipitable fraction of the urinary antigen. In one patient, desalting/dialysis with the 40 KDa MWCO, but not a 10 KDa MWCO, device resulted in partial antigen loss, suggesting that the antigen is composed of a heterogeneous population of differently-sized species, with a subpopulation between 10 and 40 KDa and another one larger than 40 KDa. In another patient, equivalent partial antigen loss was observed with all devices 2–40 KDa MWCO, suggesting fractions with antigen size <2 KDa and >40 KDa. These observations support the conclusion of variability in molecular sizes of urinary antigen, and also underscore possible differences in the nature of the urinary antigen between individuals. MWCO of resins used in desalting columns or membranes in dialysis devices are established for globular proteins and may not be accurate for polysaccharides. Even if the antigen has a high MW (>40 KDa), this does not preclude its renal excretion, since glomerular permeability of linear and asymmetric macromolecules (such as polysaccharides) is higher than that of globular proteins of equivalent MW [47]. Of note, our observations are limited to two patients and may not represent a common phenomenon. Microbial and/or host factors, such as differences in species or strains, characteristics of the milieu (*e.g.* oxygen tension, nutrient availability), presence of anti-*Aspergillus* antibodies, and antifungal use at collection time, may influence intra- and inter-patients variations in the nature of the antigen.

After the removal of the inhibitor and concentration of the sample, *Aspergillus* antigen could be detected in urine from patients with IA using a MAb 476-based lateral flow immunochromatographic assay. A small number of patient samples were included in this study to demonstrate the proof-of-concept of detection of urinary-excreted antigen in the setting of IA with a POC device. This sample size does not allow the assessment of performance characteristics. Similar devices have been developed for bacterial (*Streptococcus pneumoniae* and *Legionella pneumophila*) [48], [49] and other fungal (*Cryptococcus neoformans*) [50] urinary excreted antigens, which are also likely carbohydrates based on previous literature [34], [51], [52]. This is the first description of LFD technology for the detection of urinary *Aspergillus* Gal*f* antigens. The relatively low amounts of these antigens in urine and the inhibitory effect of this specimen which affect the analytical sensitivity are current obstacles for POC testing. Nevertheless, even with a non-optimized, lab-devised crude prototype, we detected Gal*f* antigens in clinical urine samples. The device would need to be further optimized before assessment of performance characteristics.

In summary, we have confirmed urinary excretion of *Aspergillus* Gal*f* antigen in an experimental guinea pig model and in human IA. A small molecular weight inhibitor was demonstrated in human urine. Characterization of the urinary antigen suggests a variable molecular weight polysaccharide. A prototype lateral flow device using our novel galactofuranose-reactive monoclonal antibody was shown to have the potential to detect multiple different pathogenic molds. Prospective studies will be necessary to establish the performance characteristics for this diagnostic tool and define its optimal clinical use.

## Supporting Information

Protocol S1 **Details of fungal strains and exoantigen preparations.**
(DOCX)

Protocol S2 **Details of Enzyme-linked immunosorbent assays (ELISAs).**
(DOCX)

Protocol S3 **Details of development of the lateral flow immunochromatographic assay.**
(DOCX)

Dataset S1 **Additional observations on the nature of putative urine inhibitor.**
(DOCX)

Figure S1 **Urine negatively impacts antigen-MAb476 interaction in immunoassays.** *A*, Incubation of MAb476-coated wells with urine or blocking buffer, followed by washing, prior to antigen addition; no diminution of signal: urine likely did not affect immobilized MAb476; *B*, Incubation of MAb476 in urine or PBS, followed by desalting, prior to indirect ELISA on EPA293 coated plates; no diminution of signal: urine likely did not permanently affect antigen-binding site/properties of MAb476. *C*, Incubation of EPA293 with urine or PBS, followed by desalting, prior to sandwich ELISA; no diminution of signal: urine likely did not affect the antigen or the epitope structure. *D*, Effect of urine on antigen-MAb476 complex. Additional urine incubation (1 h, 37°C, preceded and followed by washes) was introduced after the usual antigen or secondary antibody incubation step, to assess the effect of urine on, respectively, [capture-MAb476]-antigen complex or [capture-MAb476]-antigen-[MAb476-conjugate] supercomplex. These additional urine incubations led to considerable reduction in signal. Addition of urine after MAb-conjugate incubation showed worse outcome (EC_50_ comparison, *p* = 0.0058, F-test) compared to urine after EPA293 incubation.
(TIF)

Figure S2 **The putative urine inhibitor is a small (<2K Da), non-protein molecule, not affected by pH.** *A*, Desalted or dialyzed urine at different MWCOs (40 KDa, 7 KDa, 3.5 KDa and 2 KDa), used as EPA293 diluent in sELISA, all improved signal similarly compared to untreated urine. *B*, Boiling and centrifugation (B/C), acidification (pH = 5.0), alkalinization (pH = 8.0) and EDTA (10 mM) treatment of urine as a diluent for 1.1 µg/ml of EPA293 did not improve signal in sELISA; EDTA appeared to interfere slightly with assay performance. *C*, A mixture of chaotropic and kosmotropic molecules/ions (commonly present in urine) at supra-physiological concentrations (Guanidine 8, Urea 800, Ca^2+^ 10, Mg^2+^ 20 mmoles/l) appeared to be significantly inhibitory as an EPA293 diluent in comparison to PBS (PBS vs. Mix, EC_50_ comparison; *p* = 0.0005, F-test).
(TIF)
