# Supplementary material for: Detection of Urinary Excreted Fungal Galactomannan-like Antigens for Diagnosis of Invasive Aspergillosis
Source: PLoS One. 2012 Aug 10;7(8):e42736. doi: 10.1371/journal.pone.0042736 (PMC3416763; doi:10.1371/journal.pone.0042736)
Supplement: Protocol S1 — Details of fungal strains and exoantigen preparations. (DOCX) [file pone.0042736.s001.docx]

**Protocol S1: Fungal strains and exoantigen preparations**

A method described in Latgé et al. (Reference [10]) was slightly modified for ethanol-precipitation of exoantigens secreted into mycelial culture supernatants. Briefly, *Aspergillus fumigatus* Af293 conidia were grown in Aspergillus Minimal Medium (AMM) (Reference [20]) for 5-7 days (37°C; 250 rpm); the culture supernatant was filtered through sterile gauze, and the ethanol-precipitable (EP) fraction precipitated by 4X volumes of absolute ethanol. The precipitate was centrifuged at 26000*g* for 20m and washed x3 with ethanol, resuspended in water, dialyzed extensively against water and lyophilized. Other molds were grown in Sabouraud’s Dextrose Broth (SDB) with adjustments to temperature (37°C/30°C) and length of incubation (5-10 days) according to the species’ growth characteristics, and EP antigens were prepared as above. In a separate set of independent experiments in a collaborating lab (M. Feldmesser), isolates of molds (*Aspergillus* *terreus*, *Trichophyton rubrum* and *Wangiella dermatitidis*) and yeasts (*Candida albicans* and *Cryptococcus neoformans*) were grown, respectively, in AMM and SDB, and EP antigens were prepared from sterile-filtered culture supernatants using a method similar to above, but using slower centrifugation (974*g*).
