# Supplementary material for: Detection of Urinary Excreted Fungal Galactomannan-like Antigens for Diagnosis of Invasive Aspergillosis
Source: PLoS One. 2012 Aug 10;7(8):e42736. doi: 10.1371/journal.pone.0042736 (PMC3416763; doi:10.1371/journal.pone.0042736)
Supplement: Protocol S2 — Details of Enzyme-linked immunosorbent assays (ELISAs). (DOCX) [file pone.0042736.s002.docx]

**Protocol S2: Enzyme-linked immunosorbent assays (ELISAs)**

ELISAs were performed in polystyrene 96-well plates (Costar®, Corning Inc., Lowell, MA); all incubations were at 37^o^C for 1h except the color development step (30 minutes); washes were done with PBS-0.05% Tween 20 using an automated ELx405 plate washer (BioTek Instruments, Winooski, VT); color was developed with Alkaline Phosphatase (AP) substrate *p*-Nitrophenyl phosphate (pNPP, Sigma, St. Louis, MO); assays were read at 405nm.

*Indirect ELISA (iELISA).* Plates coated with 10μg/ml EPA293 in PBS and blocked with a commercial blocking agent containing plant proteins (StartingBlock® solution, Thermo Fisher) were incubated with dilutions of MAb476 in appropriate diluents (per assay design). Wells were washed and detected with AP-conjugated goat anti-IgM (SouthernBiotech Inc, Birmingham, AL) diluted 1:5000.

*Sandwich ELISA (sELISA).* Plates coated with 10μg/ml MAb476 and blocked with StartingBlock were incubated with antigen-containing samples in appropriate diluents (per assay design, PBS or blocking buffer, urine or desalted/dialyzed urine). To make the detection/secondary reagent, MAb476 was conjugated to either biotin (using EZ-Link® Sulfo-NHS-Biotin reagent; Thermo Fisher; used at 1μg/ml) or AP (using EasyLink Alkaline phosphatase Conjugation Kit; Abcam Inc., Cambridge, MA; used at 1:1000). Antigen detection was done with conjugated MAb476; biotinylated MAb476 was additionally detected with 1:5000 streptavidin-AP (SouthernBiotech).
