# Supplementary material for: Detection of Urinary Excreted Fungal Galactomannan-like Antigens for Diagnosis of Invasive Aspergillosis
Source: PLoS One. 2012 Aug 10;7(8):e42736. doi: 10.1371/journal.pone.0042736 (PMC3416763; doi:10.1371/journal.pone.0042736)
Supplement: Protocol S3 — Details of development of the lateral flow immunochromatographic assay. (DOCX) [file pone.0042736.s003.docx]

**Protocol S3: Development of the lateral flow immunochromatographic assay**

All proprietary buffers (Striping buffer, blocking buffer, conjugate block buffer and conjugate stabilizer), the colloidal gold conjugate, and physical components (Nitrocellulose [NC; HF180] membrane sourced from Millipore, adhesive backing cards, glass fiber conjugate pad and sample pad, paper waste pad, as well as the device cassettes) were obtained commercially (Millenia Diagnostics Inc., San Diego, CA), and used following the manufacturer’s instructions. Unconjugated MAb476 and a goat anti-mouse IgM (SouthernBiotech) in the striping buffer were immobilized at the test and control spots, respectively, on NC strips, followed by incubation with the blocking buffer. MAb476 was separately conjugated to colloidal gold (Millenia Diagnostics Inc.) following manufacturer’s instructions, and applied to the blocked conjugate pads. The lateral flow device (LFD) was assembled by mounting the NC membrane, conjugate pad, sample pad and absorbent pad, with appropriate overlaps, on the adhesive backing card and enclosing the system in a plastic cassette with a sample and a reaction window. Following application of 130-150μl of sample, results (appearance of spots) were read visually when NC membrane appeared dry (~30-45m). For recording results, the devices were scanned at 1200dpi, 24bit color depth, using a Brother DCP-8065DN multifunctional scanner and Brother software (Brother International, Bridgewater, NJ).
