# Supplementary material for: Detection of Urinary Excreted Fungal Galactomannan-like Antigens for Diagnosis of Invasive Aspergillosis
Source: PLoS One. 2012 Aug 10;7(8):e42736. doi: 10.1371/journal.pone.0042736 (PMC3416763; doi:10.1371/journal.pone.0042736)
Supplement: Dataset S1 — Additional observations on the nature of putative urine inhibitor. (DOCX) [file pone.0042736.s004.docx]

**Dataset S1: Additional Observations**

**Nature of putative Urine Inhibitor**

1. In the immunoassay inhibition observed with healthy urines (**Figure 2E**; VU1-6), no correlation was observed with urine pH. Other parameters tested (e.g. bilirubin, protein, nitrite, etc.) were largely normal and similar across healthy donors.
2. Dialysis or desalting through 40KDa, 7KDa, 3.5KDa and 2KDa MWCO devices abrogated the inhibitory effect of urine similarly (similar curves and EC_50_ values) (**Figure S2A**). Dilution of the inhibitory substances due to processing was not responsible for this abrogation, since initial and final volumes were not significantly different.
3. EDTA treatment of urine did not improve EPA293 signal over untreated urine; however, at 10mM or higher EDTA, it interfered slightly with the assay performance (**Figure S2B**).
4. Known chaotropes (urea, guanidine), excreted in relatively large amounts (millimolar range) in normal urine (ranges mentioned in references [24-26]), could impede the antigen-antibody interaction. However, added individually or together at physiological or supra-physiological concentrations, these chaotropes did not interfere with the assay (data not shown). We additionally tested mixes of chaotropes with more prevalent ions in urine, adding divalent cations magnesium and calcium (known kosmotropes), along with sodium, potassium, chloride and phosphates (in form of PBS). At physiological concentrations, the mix was inert, but unexpectedly, at supra-physiological concentrations (Guanidine 8, Urea 800, Ca^2+^ 10, Mg^2+^ 20 mmoles/l), the mix was significantly inhibitory as an EPA293 diluent (PBS vs. Mix, EC_50_ comparison; *p*=0.0005, F-test) (**Figure S2C**).
