# Supplementary figures and images for: Detection of Urinary Excreted Fungal Galactomannan-like Antigens for Diagnosis of Invasive Aspergillosis
Source: PLoS One. 2012 Aug 10;7(8):e42736. doi: 10.1371/journal.pone.0042736 (PMC3416763; doi:10.1371/journal.pone.0042736)

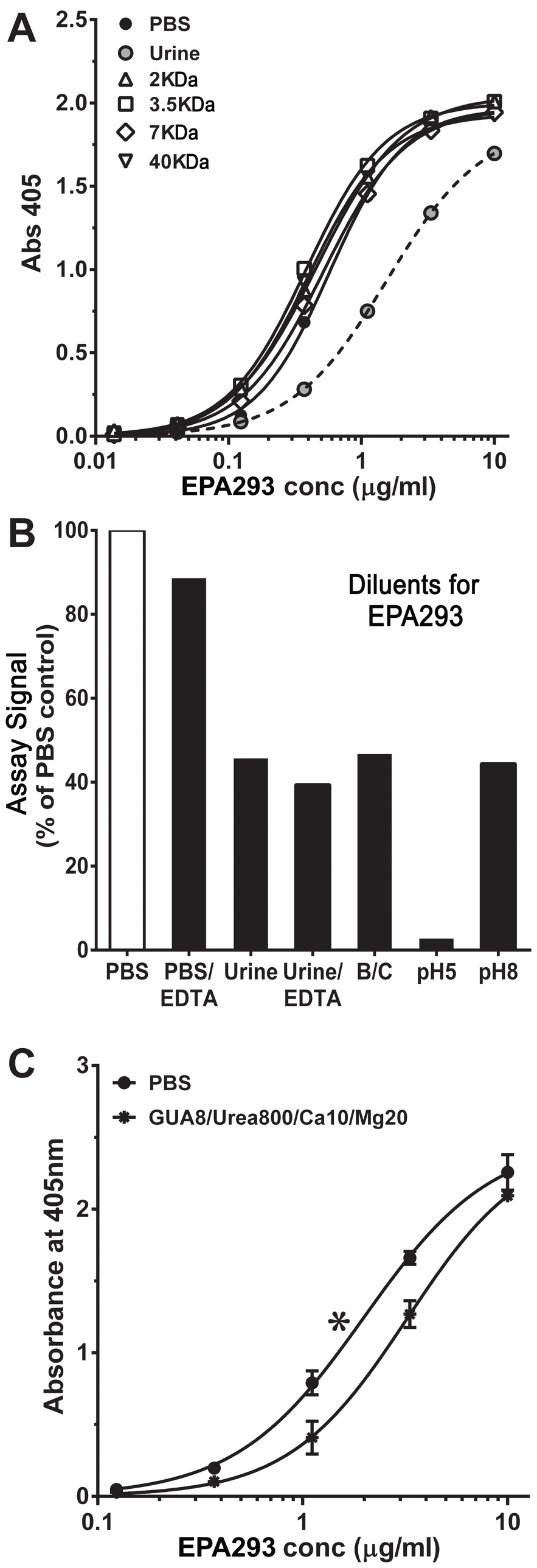

Supplement: Figure S2 — The putative urine inhibitor is a small (<2K Da), non-protein molecule, not affected by pH. A, Desalted or dialyzed urine at different MWCOs (40 KDa, 7 KDa, 3.5 KDa and 2 KDa), used as EPA293 diluent in sELISA, all improved signal similarly compared to untreated urine. B, Boiling and centrifugation (B/C), acidification (pH = 5.0), alkalinization (pH = 8.0) and EDTA (10 mM) treatment of urine as a diluent for 1.1 µg/ml of EPA293 did not improve signal in sELISA; EDTA appeared to interfere slightly with assay performance. C, A mixture of chaotropic and kosmotropic molecules/ions (commonly present in urine) at supra-physiological concentrations (Guanidine 8, Urea 800, Ca2+ 10, Mg2+ 20 mmoles/l) appeared to be significantly inhibitory as an EPA293 diluent in comparison to PBS (PBS vs. Mix, EC50 comparison; p = 0.0005, F-test). (TIF) [file pone.0042736.s006.tif]
